# Transcriptional activators YAP/TAZ and AXL orchestrate dedifferentiation, cell fate, and metastasis in human osteosarcoma

**DOI:** 10.1038/s41417-020-00281-6

**Published:** 2021-01-06

**Authors:** Salah-Eddine Lamhamedi-Cherradi, Sana Mohiuddin, Dhruva K. Mishra, Sandhya Krishnan, Alejandra Ruiz Velasco, Amelia M. Vetter, Kristi Pence, David McCall, Danh D. Truong, Branko Cuglievan, Brian A. Menegaz, Budi Utama, Najat C. Daw, Eric R. Molina, Rafal J. Zielinski, John A. Livingston, Richard Gorlick, Antonios G. Mikos, Min P. Kim, Joseph A. Ludwig

**Affiliations:** 1 Sarcoma Medical Oncology Department, The University of Texas MD Anderson Cancer Center, Houston, TX USA; 2 Division of Pediatrics, The University of Texas MD Anderson Cancer Center, Houston, 77030 TX USA; 3 Division of Thoracic Surgery, Department of Surgery, Houston Methodist Hospital, Houston, 77030 TX USA; 4 Department of Surgery, Breast Surgical Oncology, Baylor College of Medicine, Houston, 77030 TX USA; 5 Optical Microscopy Facility, Rice University, Houston, 77030 TX USA; 6 Bioengineering Department, Rice University, Houston, 77030 TX USA; 7 Experimental Therapeutics Department, The University of Texas MD Anderson Cancer Center, Houston, 77005 TX USA

**Keywords:** Sarcoma, Biomarkers

## Abstract

Osteosarcoma (OS) is a molecularly heterogeneous, aggressive, poorly differentiated pediatric bone cancer that frequently spreads to the lung. Relatively little is known about phenotypic and epigenetic changes that promote lung metastases. To identify key drivers of metastasis, we studied human CCH-OS-D OS cells within a previously described rat acellular lung (ACL) model that preserves the native lung architecture, extracellular matrix, and capillary network. This system identified a subset of cells—termed derived circulating tumor cells (dCTCs)—that can migrate, intravasate, and spread within a bioreactor-perfused capillary network. Remarkably, dCTCs highly expressed epithelial-to-mesenchymal transition (EMT)-associated transcription factors (EMT-TFs), such as ZEB1, TWIST, and SOX9, which suggests that they undergo cellular reprogramming toward a less differentiated state by coopting the same epigenetic machinery used by carcinomas. Since YAP/TAZ and AXL tightly regulate the fate and plasticity of normal mesenchymal cells in response to microenvironmental cues, we explored whether these proteins contributed to OS metastatic potential using an isogenic pair of human OS cell lines that differ in AXL expression. We show that AXL inhibition significantly reduced the number of MG63.2 pulmonary metastases in murine models. Collectively, we present a laboratory-based method to detect and characterize a pure population of dCTCs, which provides a unique opportunity to study how OS cell fate and differentiation contributes to metastatic potential. Though the important step of clinical validation remains, our identification of AXL, ZEB1, and TWIST upregulation raises the tantalizing prospect that EMT-TF-directed therapies might expand the arsenal of therapies used to combat advanced-stage OS.

## Introduction

Osteosarcoma (OS) is the most common bone sarcoma and a leading cause of cancer deaths in adolescents and young adults [1, 2]. Despite surgical advances that have improved local control rates, the systemic therapies used to treat OS today rely on the same four drugs used since the mid-1980s [3–5]; expectedly, the 5-year survival rate from metastatic OS has not appreciably changed since that era and remains at 20–30% [5–7]. Since traditional cytotoxic chemotherapies and newer immunotherapies seem unable to eradicate genetically heterogeneous OS cells, our research focused its attention at the sentinel feature of high-grade OS tumors, which is their composition of poorly differentiated or undifferentiated stem-like tumor cells that appear in various states of partial osteoblastic, chondroblastic, and adipogenic commitment [8].

Except for the presence or absence of metastatic disease at diagnosis, tumor grade—which gauges how well tumors resemble their normal tissue counterpart histologically—is the single most important predictor of a patient’s clinical course and outcome [1]. Consistently, high-grade poorly differentiated sarcomas are significantly more proliferative, invasive, and metastasis-prone than their low-grade counterparts. In light of the broad sweeping effect that tumor grade and differentiation have upon cell invasion and metastasis, it comes as no surprise that many cancer hallmarks defined by Hanahan and Weinberg are intricately linked to cell fate [9, 10]. Among the hallmarks of interest to our current studies, chemoresistance, genome instability, migratory potential, and invasiveness have all been associated with cancer stem cells (CSCs) [11, 12]. The relationship between cell fate, differentiation, and cancer hallmarks in OS is far less studied, and it remains to be determined whether dedifferentiated OS cells become, or just phenocopy, CSCs.

To help explore this relationship, our research draws from recent efforts by tissue engineers to fabricate laboratory-grown tendons, muscle, and bone from normal mesenchymal stem cells (MSCs) pushed toward distinct cell fates in response to growth factors and biophysical forces [13–17]. Using MSC differentiation as the reference standard, we can observe how disturbed cell signaling pathways might invoke the jumbled pattern of differentiation observed in high-grade OS. As a byproduct of the OS’s hypothesized inability to sense and respond to microenvironmental cues that should otherwise have steered them toward a mature osteoblastic cell fate, one would predict that OS contains an undifferentiated, genetically defective MSC-like cell population (perhaps CSCs) that continues to produce daughter cells capable of partial trilineage differentiation [18–22]. Though limited data exist in OS, evidence from other cancer types suggests that this small minority of stem-like tumor cells are responsible for tumor propagation and, ultimately, more likely to escape the primary tumor (PT), circulate in the bloodstream as circulating tumor cells (CTCs), and metastasize to the lungs [23].

To determine whether those hypotheses were correct, we faced two hurdles. First, because the differentiation from MSCs into mature osteoblasts is inextricably linked to the surrounding tissue architecture and matrix stiffness, there was concern that non-physiological tissue culture plasticware, devoid of differentiation cues, would obscure any effect of differentiation upon cell phenotype, or worse, lead to incorrect conclusions. Second, and perhaps more problematic, because CSCs are rare (perhaps less than one cell in a thousand), there was no tractable solution to identify and profile a sufficient number of CSCs within a native tumor as it exists in vivo.

To overcome those challenges, we turned to a novel ex vivo model that we had previously optimized to interrogate human lung cancer [24–30]. This decellularized rat lung model (acellular lung (ACL)), also described as a four-dimensional lung metastasis model, provides OS cells with the full spectrum of biological substrates needed for tumor growth within a three-dimensional (3D) collagen network enmeshed with a decellularized vascular network suitable for perfusion of nutrient-enriched media. In a flow perfusion bioreactor that circulates media throughout the decellularized residual vascular network, tumor cells propagate to form full-fledged macroscopic tumors that recapitulate much of the complex in vivo tumor heterogeneity. While fostering the formation of large ACL-embedded tumors used to assess intratumoral heterogeneity, an added advantage of this model was the ability to study CTCs, a distinct subset of cells capable of completing the multi-step process required to form metastatic tumors.

As will be shown, these so-called derived CTCs (dCTCs) expressed proteomic markers previously reported in invasive OS and, surprisingly, shared numerous genes linked to the epithelial-to-mesenchymal transition (EMT) that occurs in epithelial malignancies [31, 32]. The finding that dCTCs expressed genomic and proteomic features previously reported for EMT raised the prospect that OS dCTCs stemmed from a population of cells had either failed to differentiate or had epigenetically converted to a less differentiated, more invasive cell type. As sarcomas are mesenchymal by their very definition and generally lack epithelial features, the EMT terminology would not make sense linguistically.

Nevertheless, the essence of EMT, which is the rejuvenation of mature cells toward a less differentiated, more pluripotent stem-like state, has been reported to occur in a wide range of non-epithelial tissues, including gliomas, leukemias, and sarcomas of various subtypes [33–35]. In this latter scenario—and routinely throughout this text—we describe this EMT-like phenomenon more appropriately as dedifferentiation, defined as the natural acquisition of a less differentiated uphill state on the proverbial Waddington epigenetic landscape. Apart from this difference in terminology, EMT and dedifferentiation share much in common. We note that, within the ACL model and other engineered tumor microenvironments (TMEs), EMT and dedifferentiation occur naturally in response to EMT-associated transcription factors (EMT-TFs) and TME cues, as opposed to cell reprogramming, a closely related process that invokes pluripotency by introducing exogenous TFs into the cell.

Recently, many roles for Yes-associated protein (YAP) and TAZ (encoded by WWTR1) as main mediators of the Hippo pathway have been described in (a) regulating organ size [36]; (b) promoting tumor initiation, progression, metastasis [37, 38], and EMT [39]; and (c) reprogramming cancer cells into CSCs [40–42]. The activation of the YAP/TAZ protein in high tensegrity microenvironments favors YAP/TAZ nuclear shuttling from the cytoplasm to the nucleus, where it partners with TEAD as a transcriptional coactivator of several genes, including CTGF and AXL, to promote cell proliferation and survival programs [43–45].

In the current work, our use of the ACL model afforded the ability to derive a purified population of dCTCs quickly. Genomic and proteomic interrogation of these cells provides an initial glimpse of the cellular changes required to invoke the dedifferentiation and invasive capacity of OS cells. As will be demonstrated, several proteins classically associated with EMT, such as AXL, were significantly overexpressed in dCTCs and a highly metastatic MG63.2 OS cell line. That AXL inhibition reduced cell proliferation, invasion, and metastasis suggests the EMT pathway—as interpreted as dedifferentiation in sarcoma—may provide new clues to combat an aggressive bone cancer that to date have remained elusive.

## Materials and methods

### Regulatory

The animal studies have been approved by the Institutional Animal Care and Use Committee at the Houston Methodist Research Institute (Protocol#AUP-0716-0037) and MD Anderson Cancer Center (MDACC; Protocols #00001903-RN00 and #00001904-RN00).

### Cell culture and lung harvest

The isogenic metastatic MG63.2 cell line is derived from a non-metastatic MG63 cell line through serial passage in nude mice via intratibial injections [46, 47]. The parental MG63 was first established from a 14-year-old OS patient [48]. Both MG63, MG63.2, and OS-D cell lines (that were derived also from OS patient specimens were gifted from Dr. Kleinerman’s Laboratory (MDACC, Department of Pediatrics, Houston, TX). OS-D, MG63, and MG63.2 parental or luciferase-expressing cells were grown in high glucose Dulbecco’s modified Eagle’s medium (Hyclone, USA) supplied with 10% fetal bovine serum (Gemini, USA), and 1× Penicillin–Streptomycin–Amphotericin (Life Technology, USA) in flasks. Cells were tested twice per year for mycoplasma contamination using the MycoAlert Detection Kit (Lonza Group Ltd.) according to the manufacturer’s protocol, and cell line identity was validated using short-tandem repeat fingerprinting with an AmpFLSTR Identifier Kit as previously described [49]. To grow the OS-D cells on the ACL model, the Lung–Heart block from a Sprague Dawley rat (Envigo) was harvested, and the pulmonary artery was cannulated as previously described [25]. Additional details about lung decellularization and perfusion are provided in Supplementary Methods.

### **OS cell culture in the** ex vivo **lung model**

**Fig. 1:**
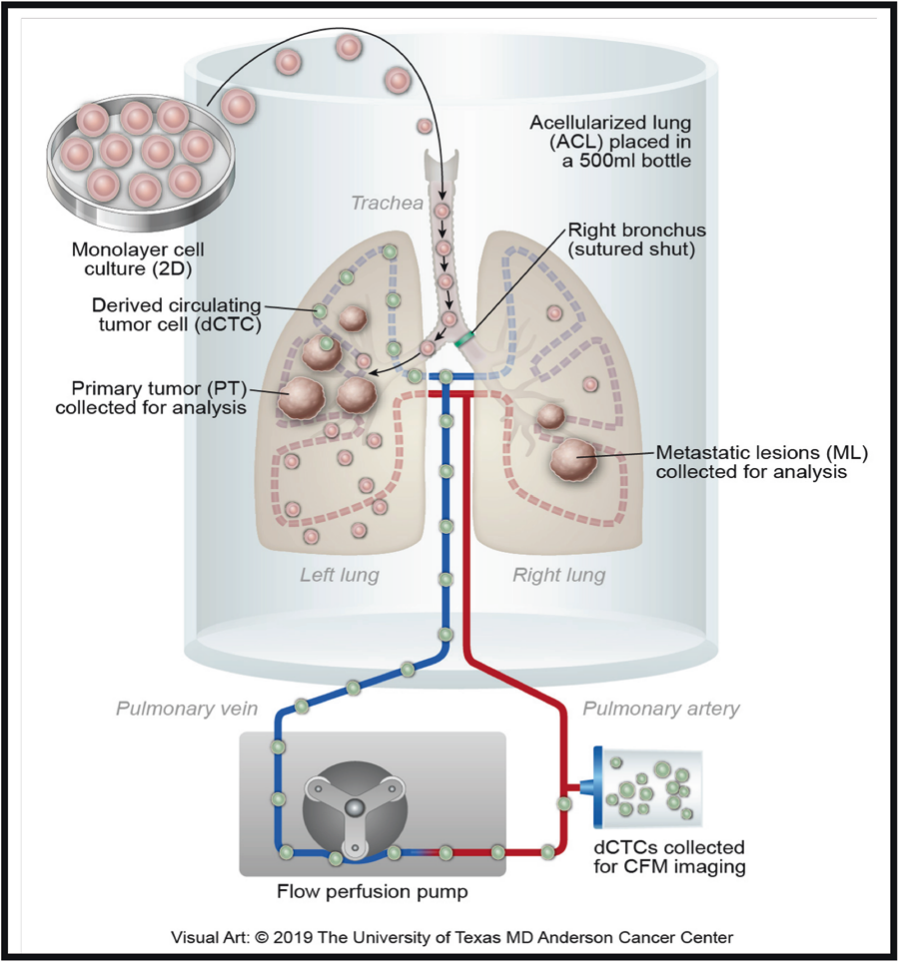
Diagrammatic representation of the acellular lung (ACL) model. Right lung lobes were tied by silk, and 2D cells were seeded to the left lung lobe through the trachea. As the nutrient media perfuse to the lung, tumor nodules on left lobes grow and form dCTCs that intravasate to the vasculature, survive in circulation, and enter the right lobes through the pulmonary artery (PA) and forms metastatic lesions.

For the ACL cell culture, a customized bioreactor with three outlets in the cap (one for the pulmonary artery, one for trachea, and one for outflow) was created and set up in a cell culture incubator along with a pump and 10 feet Tygon tubing for oxygenation (Fig. 1). The ACL was then connected in the bioreactor through the pulmonary artery cannula. The trachea was connected to the tracheal cannula while the outflow was connected to an oxygenator. The cell culture media was run through the lung by a pump at a perfusion pressure of 6 ml/min. Additional details are provided in Supplementary Methods and as described previously [30].

### Treatment of OS-D cells on ACL model with doxorubicin

The ACL model was seeded with OS-D cells and grown for 7 days. On day 7 and during 24 h, 3 μM of doxorubicin (Sigma, USA) was infused in the treatment bioreactor, while the control group received a similar volume of ethanol. Then the media was changed, and the cells were grown for the next 2 days. Additional details are provided in Supplementary Methods.

### Histology and immunohistochemistry (IHC) analyses

A rat lung lobectomy was performed on different days, and the ACL tissue was fixed in 10% formalin, embedded in paraffin, and then sliced in 4 μm sections for hematoxylin–eosin staining. We also performed IHC analyses of the formalin-fixed paraffin-embedded sections for Ki-67 and caspase-3, as described previously [30]. Expert board-certified pathologists examined the stained slides, and the images were captured using a microscope (Evos, Mill Creek, WA). We calculated the proliferation index (Ki-67) and apoptotic index (caspase-3) using ten random pictures taken at ×40 magnification for each specimen stain, and both the total number of cells and the number of positive cells were counted to yield the percentage of positive cells expressing those proteins. More details are provided in Supplementary Methods.

### Western blot profiling

Protein lysis from cells or tumors using RIPA buffer, protein quantification by Bradford assay, and western blots were performed as previously described by our group [50]. More details are provided in Supplementary Methods.

### MG63 and MG63.2 OS in vivo models and pulmonary metastasis assessment

Animal studies were conducted following the University of Texas MDACC Committee on Animal Care protocol (Protocol #00001904-RN00). Additional details about experimental lung metastasis experiments are provided in Supplementary Methods.

### Statistical analyses

Statistical analyses comparing two groups were performed using the GraphPad Prism 7 software (GraphPad Software Inc.) selecting the unpaired *t* test. A value of *p* < 0.05 was considered statistically significant.

## Results

### An ex vivo ACL model of OS for anticancer drug testing

As an alternative to in vitro preclinical models that frequently overestimate the efficacy of anticancer therapeutics, we chose a simple ACL model that preserves critical tumor-promoting elements of the metastatic OS microenvironment, including the native lung extracellular matrix, complex 3D tissue architecture, and an intact vascular network that enables mass transport of nutrients and metabolic waste byproducts. Because the acellularized capillary network permeates the ACL, a bioreactor can deliver oxygenated, nutrient-rich, cell culture media to tumor cells, thereby fostering the development of macroscopic tumors. To test whether OS cells would survive, proliferate, and metastasize within this ex vivo ACL model, primary human OS-D cells were introduced into the left lung via the trachea. As described extensively in the “Methods” and Supplemental Methods sections, before injecting the cells, the right main stem bronchus is sutured shut (Fig. 1). In that configuration, tumors develop in the left lung by direct extension. However, they can only reach the right side when cells migrate from the left-sided lung tumors, intravasate into the bioreactor-perfused pulmonary vessels as dCTCs, and lodge within the contralateral lung capillary network (i.e., lung-to-lung metastases).

OS-D tumor nodules formed in the ACL within 3 days, as shown macroscopically (Supplemental Fig. 1A) and microscopically at various magnifications (Supplemental Fig. 1B, C). As a standard chemotherapy used to treat OS, doxorubicin (3 μM) was used to evaluate PT regression beginning on day 7 (Supplemental Fig. 2A–D). Doxorubicin treatment reduced the formation of tumor nodules (Supplemental Fig. 2A–D) compared to the untreated control group (24.3 ± 9.1 vs. 833 ± 130, *p* < 0.0001). Similarly, there were significantly more metastatic lesions with the untreated control group (Supplemental Fig. 1D) compared to the model treated with doxorubicin (Supplemental Fig. 2D) (147 ± 61 vs. 0, *p* < 0.0001).

To evaluate doxorubicin’s effect, respectively, upon cell proliferation and apoptosis, each specimen was IHC stained separately with an antibody against Ki-67 and caspase-3. The percentage of positive cells was counted and averaged to yield the proliferation and apoptotic indices. Our analysis showed a moderate difference in the proliferation index (Ki-67) in the control group (Supplemental Fig. 2E) compared to the PT treated with doxorubicin (Supplemental Fig. 2F) (81.6 ± 5.1% vs. 69.3 ± 18.3%, *p* = 0.057) (Supplemental Fig. 2I). The apoptotic index was expectedly much higher in the doxorubicin-treated OS model (Supplemental Fig. 2H) compared to the untreated control group (Supplemental Fig. 2G, J; 78.8% vs. 7.2%, *p* < 0.01). Moreover, there were significantly more metastatic lesions in the untreated control group than the model treated with doxorubicin (Supplemental Fig. 2K, L; 147 ± 61 vs. 0, *p* < 0.0001). To further assess the sensitivity of doxorubicin in the ACL model, dCTC were more resistant to doxorubicin at 48 h than 2D cells (Supplemental Fig. 2M, N; *p* < 0.0001). There was no significant difference in cell number between control and doxorubicin treatment (Supplemental Fig. 2N; *p* = 0.75) after 48 h of treatment. Though other chemotherapies remain to be tested, our results suggest that the ex vivo ACL OS model exhibits in vivo-like sensitivity to chemotherapy.

### The ACL model facilitates the study of OS metastasis

As the ACL was connected in the bioreactor through the pulmonary artery cannula (Fig. 1), within 7 days of tumor formation, thousands of dCTCs had entered the culture media perfused through the capillary network. Intermittently every 48 h, dCTCs were harvested from the conditioned medium circulating through the capillary network, which presented our team with an unexpected opportunity to study lung-to-lung hematogenous OS spread using an ex vivo system free of white blood cell contaminants. Examination of the right lung revealed metastatic nodules (Supplemental Fig. 1D), and two methods were used to assess the metastatic potential of OS-D cells in our ex vivo ACL model. By tying off the right bronchus, the first method models the full spectrum of chronological events required for lung-to-lung metastases: (a) tumor cell motility within the left-sided pulmonary nodules, (b) dCTC intravasation into the left lobe lung vasculature, (c) dCTC survival within the circulating culture media, and (d) eventual survival and extravasation of dCTC into the contralateral right lung parenchyma (Supplemental Fig. 1D). The second method, directly measures the latter two events (e.g., anoikis and extravasation) and, importantly, begins with OS-D cells directly trypsinized from 2D monolayers that are injected into the pulmonary artery (Supplemental Fig. 1E–H).

### dCTCs upregulate receptor tyrosine kinases (RTKs) linked to metastasis

**Fig. 2:**
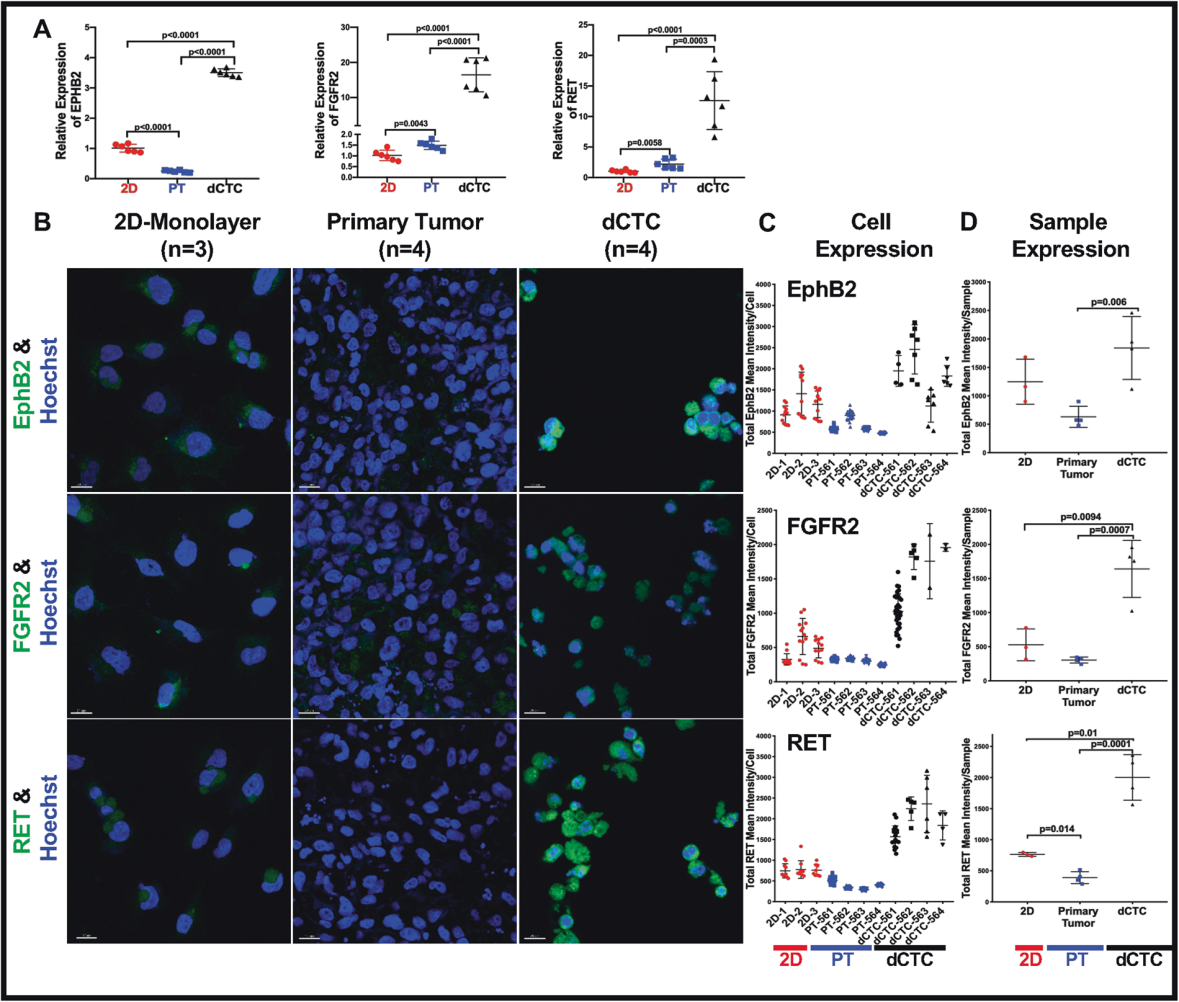
OS-D dCTCs upregulate receptor tyrosine kinases (RTKs) linked to metastasis. **A** CTCs derived from the ex vivo 4D ACL model have significantly higher EPHB2, FGFR2, and RET gene expression, as compared to respective primary tumor nodules formed on this model and OS-D cells grown as 2D-monolayer. Columns represent the mean values of *n* = 3 experiments, and bars represent standard deviations. **B** Representative EphB2, FGFR2, and RET immunofluorescence confocal microscopic staining and quantification, **C** within the single cell, or **D** the averaged OS-D-2D, PT, and dCTC samples. 20 μm bars are shown. Scatter plots represent the mean value of three experiments for OS-D 2D-monolayer cultures and four experiments for the PT and dCTC. Bars represent standard deviations. PT primary tumor, dCTC derived circulating tumor cell.

Several RTKs play an essential role in the regulation of stemness, invasion, and metastasis [51]. Therefore, we hypothesized our OS dCTCs would express a RTK pattern similar to human CTCs that emanate from OS xenograft models. Focusing initially on a subset of previously reported preclinical biomarkers [32], we measured the expression of EPHB2, FGFR2, and RET in OS-D cells taken from monolayers cultures (2D), ACL PT, and ACL dCTC (Fig. 2). Reverse transcriptase polymerase chain reaction (RT-PCR) data from three independent ACL experiments revealed a statistically significant upregulation of EPHB2, FGFR2, and RET mRNA in dCTC as compared to monolayer and PT cultures (Fig. 2A). To better delineate the location and nuclear-to-cytoplasmic distribution of the three protein biomarkers, we used confocal microscopy (Fig. 2B–D) to evaluate the cells or tissues at the single-cell (Fig. 2C) and averaged sample levels (Fig. 2D). A detailed description of the approach used for cell visualization, analysis, segmentation, and interpretation as performed using semi-automated Imaris software algorithms is described in Supplemental Methods section. FGFR2 and RET were significantly upregulated in PT formed by OS cells, while EPHB2 was significantly downregulated compared to 2D OS cells (*p* < 0.0001, Fig. 2A). These data collectively indicate that the ACL TME significantly alters the RTK profiles that govern the OS metastatic cascade.

### The Hippo pathway, YAP/TAZ, and AXL are enhanced in dCTCs

**Fig. 3:**
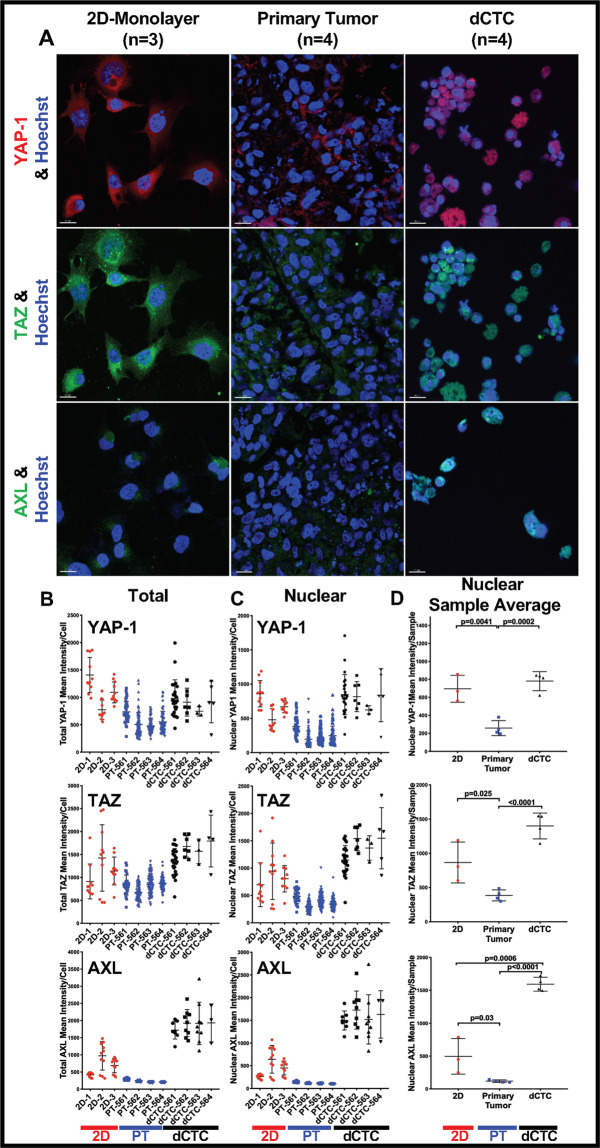
The Hippo pathway, YAP/TAZ, and AXL are enhanced in OS-D dCTCs. **A** Representative YAP-1, TAZ, and AXL immunofluorescence confocal microscopic staining and quantification, **B** within the total single cell, **C** the nuclear single cell, and **D** the averaged OS-D-2D, PT, and dCTC samples. 20 μm scale bars are shown. Scatter plots represent the mean value of three experiments for OS-D 2D-monolayer cultures and four experiments for the PT and dCTC. Bars represent standard deviations. PT primary tumor, dCTC derived circulating tumor cell.

Healthy and cancer cells alike sense their microenvironment through soluble, physical, and mechanical cues by translating them into biochemical signals that regulate cellular behavior, including differentiation and metastasis. In that process, the YAP (Yes-associated protein) and TAZ (Transcriptional Co-Activator with PDZ-Binding Motif) paralogs serve as important transcriptional coactivators that dictate when and how MSCs transition into cells forming bone, fat, cartilage, or muscle. To determine whether YAP/TAZ activation, and downstream effectors such as AXL, contributed to the dCTC phenotype [52–54], we compared the expression of YAP/TAZ and AXL within OS-D cells cultured upon 2D monolayer, within the ACL PT, and among the dCTCs. We used confocal microscopy to quantify each protein’s subcellular localization in individual cells because the epigenetic effects of activated YAP/TAZ proteins necessitate TEAD binding and transmigration into the nucleus (Fig. 3). Consistent with published reports, total and nuclear (i.e., active) YAP/TAZ levels are significantly elevated when OS-D cells are cultured on 2D cultureware that are several orders of magnitude stiffer than human lung tissue (Fig. 3). Compared to the PT from which they emanated, dCTCs expressed higher YAP and TAZ, which were predominantly confined to the cell nucleus (Fig. 3B–D and Supplemental Fig. 3A).

Even more striking was the upregulation in dCTCs of AXL, an RTK known to mediate YAP/TAZ oncogenic effects [54, 55], OS metastasis [32], and chemoresistance in several cancer types [56–60]. We quantified AXL expression by RT-PCR (Supplemental Fig. 3B), immunofluorescence, and confocal analyses (Fig. 3A and B–D lower panels). These findings indicate that the AXL/YAP/TAZ axis may have a critical role in OS cell fate and metastasis and serve as a viable drug target.

### The expression of EMT-TFs suggests that dCTCs may have undergone dedifferentiation

As dCTCs strongly express proteins linked to metastasis, we questioned whether YAP/TAZ, AXL, and other TFs induced epigenetic changes favoring what would be classically described as EMT in epithelial malignancies. As elaborated in the “Introduction,” the epigenetic processes associated with dedifferentiation and EMT were suspected to be quite similar in OS.

**Fig. 4:**
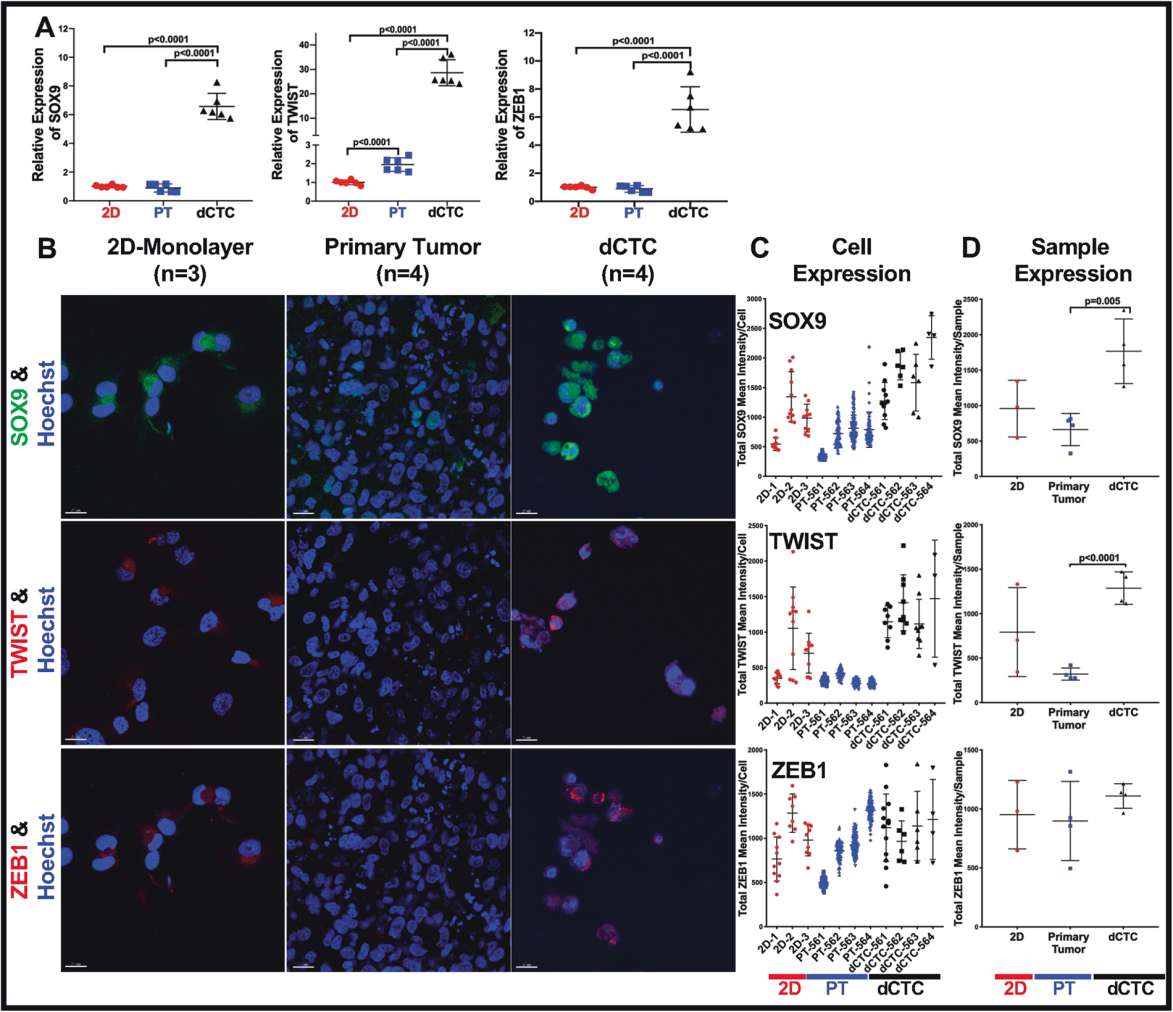
The expression of EMT-associated TFs suggests that OS-D dCTCs may have undergone dedifferentiation. **A** As assessed by RT-PCR, dCTCs derived from the ex vivo 4D ACL model have significantly higher SOX9, TWIST, and ZEB1 gene expression, as compared to respective primary tumor nodules formed on this model and OS-D cells grown as 2D monolayer. Columns represent the mean values of *n* = 3 experiments, and bars represent standard deviations. **B** Representative SOX9, TWIST, and ZEB1 immunofluorescence confocal microscopic staining and quantification, **C** within the single cell, or **D** the averaged OS-D-2D, PT, and dCTC samples. 20 μm scale bars are shown. Scatter plots represent the mean value of three experiments for OS-D 2D-monolayer cultures and four experiments for the PT and dCTC. Bars represent standard deviations. PT primary tumor, dCTC derived circulating tumor cell.

To investigate this, we employed RT-PCR and immunofluorescence to analyze previously described cell stemness markers (SOX9, TWIST, ZEB1, SNAI2, N-Cadherin (N-CDH)). Compared to the PT, dCTC upregulated SOX9 and TWIST by 6–30-fold (Fig. 4A–D). ZEB1, known to transcriptionally repress epithelial genes responsible for cell polarity and migratory potential, was also considerably elevated at the RNA, but not at protein, as compared to 2D and PT [61]. This result might be due to its heterogeneous expression within the replicates of each sample (Fig. 4B–D) or due to the differential effects of ZEB1 in sarcomas vs. carcinomas.

The RT-PCR analyses also showed a significant upregulation of Snail2 (SNAI2) in the dCTCs compared to PT (Supplemental Fig. 4A, *p* < 0.0001). N-CDH, a protein often expressed in mesenchymal tissues, was also upregulated in dCTCs (Supplemental Fig. 4B–D, *p* = 0.0004), a finding confirmed by immunofluorescence and quantified by Imaris image analysis. Collectively, this data suggests that dCTC have acquired stem cell features that may have aided their dissemination into the perfused vascular space.

### AXL inhibition attenuates the proliferation, migration, and metastasis of OS cells

To investigate whether AXL was contributing to OS cell growth, migration, and metastasis, we treated in vitro an isogenic pair of metastatic and non-metastatic human OS cell lines (MG63.2/MG63) with a small molecule inhibitor of AXL (AXLi, SGI-7079) [47]. The metastasis-prone OS MG63.2 cells, as compared to parental MG63 cells, highly expressed AXL by immunofluorescence confocal imaging analysis (Supplemental Fig. 5A). In addition, MG63.2 cells were more sensitive to AXL inhibition compared to MG63 cells, as shown through a proliferation cell-based assay (Supplemental Fig. 5B). To verify in vitro on-target AXLi effects, we performed a western blot analysis, demonstrating that SGI-7079 blocked exogenous stimulation by its cognate ligand (Gas6; Supplemental Fig. 5C, D) and slowed in vitro migration of MG63.2 cells as compared to parental MG63 cells (Supplemental Fig. 6). Therefore, the different expression of AXL between MG63 and MG63.2 cells might be the consequences of these cells responding variably to AXLi, through their proliferation or migration.

**Fig. 5:**
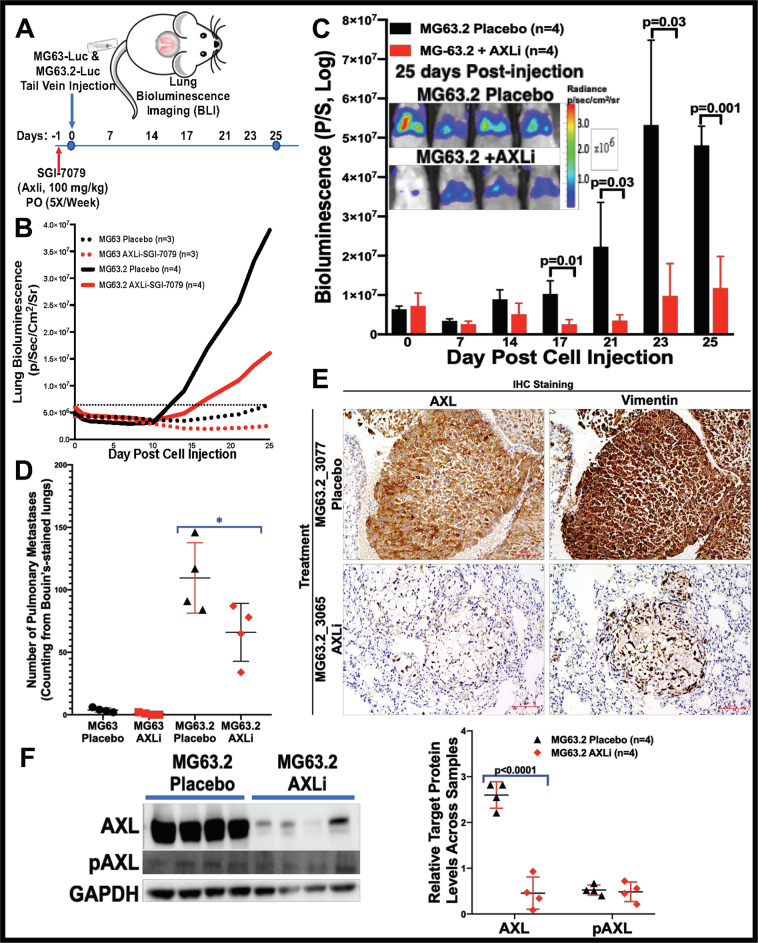
AXL inhibition attenuates the metastasis of MG63.2 OS cell lines. **A** Preclinical experiment design. **B** Monitoring OS tumor growth in lung and SGI-7079 (AXL inhibitor, AXLi) treatment response after tail vein injection of MG63/MG63.2-luciferase cell lines in immunodeficient mice. The luminescent intensity of photons emitted from each tumor from control and AXLi-treated mice after injections with 5 × 10^5^ luciferase- MG63/MG63.2 cells. **C** Bioluminescent imaging of mice 25 days post-tail vein injection of 5 × 10^5^ MG63.2-expressing luciferase during AXLi and placebo treatments. **D** Quantification of pulmonary metastatic nodules in MG63 and MG63.2 xenograft mice after AXLi and placebo treatments. **E** IHC stains for AXL and vimentin in lung tissues of MG63.2 xenograft mice after AXLi or placebo treatments. **F** Western blot analyses of AXL and pAXL expression in lung tissues of MG63.2 xenograft mice after AXLi or placebo treatments (left panel). Normalized AXL and pAXL expression relative to GAPDH in lung tissues of MG63.2 xenograft mice after AXLi or placebo treatments (right panel) with unpaired two-tailed Student’s *t* test statistical analyses; bars show mean ± SD.

Next, to test whether AXLi would affect OS metastasis in vivo, the same isogenic pair of cell lines, co-expressing luciferase, were injected via tail vein into immunocompromised NSG mice (Fig. 5A). Lung metastases were monitored via bioluminescence in live animals using the IVIS Spectrum in vivo imaging system, and representative images of Bouin’s solution-stained lungs were analyzed after 4 weeks of AXLi. The bioluminescence images were analyzed using the Living Image software (PerkinElmer). AXL blockade of MG63.2 cells by SGI-7079 significantly reduced tumor bioluminescence (Fig. 5B, C) and the number of pulmonary metastases (Fig. 5D). AXLi significantly reduced the AXL expression levels but not of pAXL or vimentin, which is considered as an exclusive marker on OS CTC [62], as shown in Fig. 5E, F and Supplemental Fig. 7, shown by IHC and western blotting. These results indicate a direct role for AXL in OS proliferation, migration, and metastasis.

## Discussion

To study OS metastases at the main site responsible for cancer deaths, we demonstrate the ability to grow OS cells in an innovative native ACL matrix model (Fig. 1). Like lung cancer cells placed through the trachea [63], OS cells spread throughout the lung to form distinct, rapidly growing nodules that can reach ≥1 cm in size. This capacity to form macroscopic tumors is facilitated by a bioreactor and paired residual lung capillary network that provides a biological conduit to nourish growing tumors with oxygenated, nutrient-rich cell culture media while simultaneously eliminating waste byproducts. As opposed to monolayer and spheroid cell culture models that lack 3D architecture and contain hypoxic cores that affect drug sensitivity, the ACL model provided a unique opportunity to study chemotherapy response that better resemble the human tumor counterparts. As highlighted by our results, the model also yielded abundant dCTCs, which allowed us to investigate the cell fate changes that enabled their intravasation.

The ACL model yielded striking differences in chemosensitivity, using doxorubicin, a standard treatment for OS. Like lung cancer cells treated with cisplatin [29], the OS cells grown in the ACL showed a significant increase in apoptosis with a single treatment of doxorubicin compared to untreated control samples. Interestingly, dCTCs were also resistant to doxorubicin (Supplemental Fig. 2), a phenomenon reported in lung cancer dCTCs treated with cisplatin [29].

**Fig. 6:**
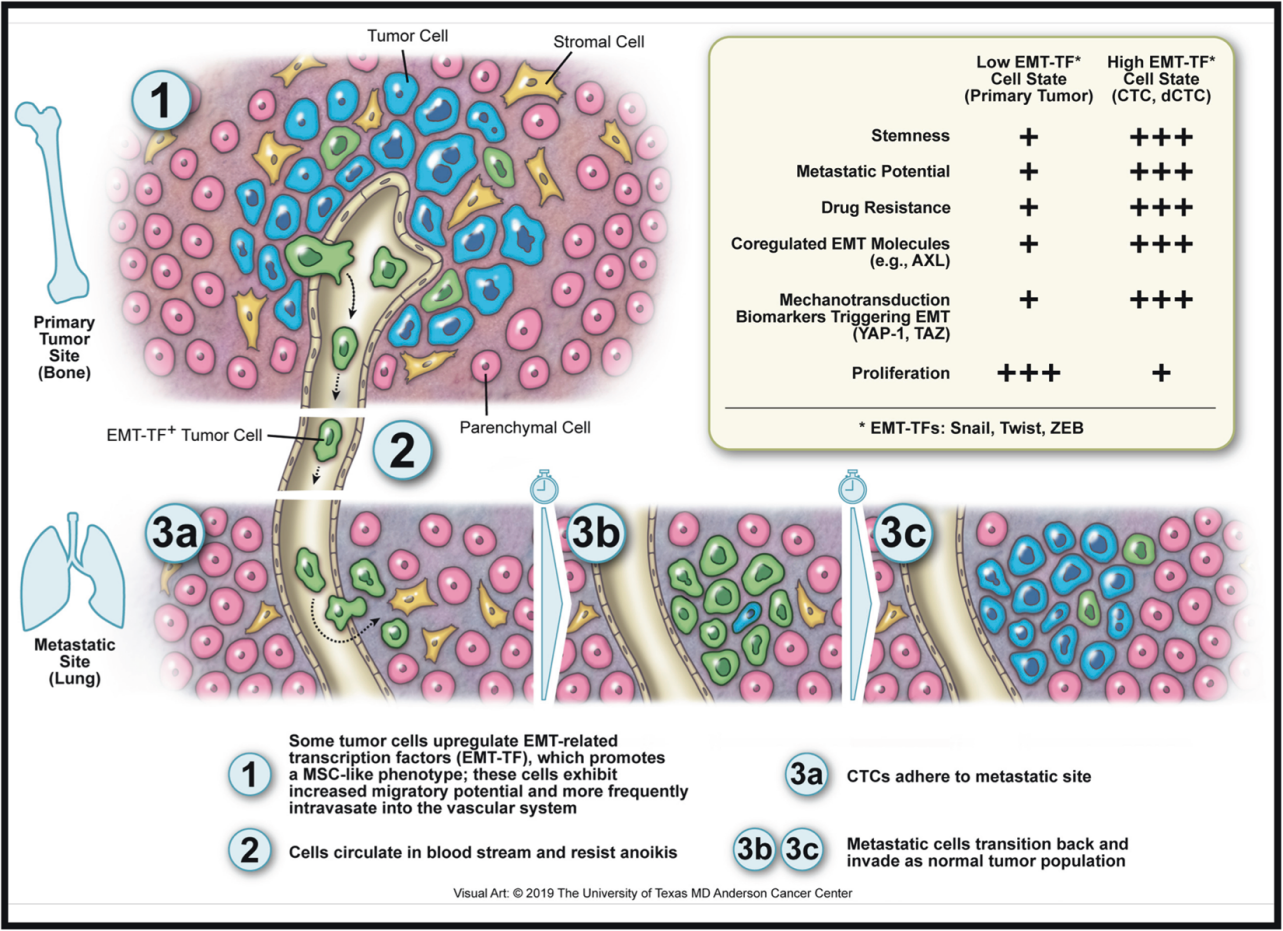
The model describing how EMT-like related machinery promotes OS dedifferentiation and enhanced metastatic potential. Metastatic cascade occurs within a small subset of primary tumor cells that acquire the ability to migrate, intravasate into the bloodstream, resist anoikis, and survive at distant tissue sites, like the lung. CTC circulating tumor cell, dCTC derived circulating tumor cell.

With ligation of the right main stem bronchus, the ACL model provided a unique opportunity to simulate—ex vivo—the metastatic cascade typically observed in living organisms. OS cells migrate to the lung only after well-described steps that include intravasation from primary osseous sites into circulation as CTCs, resistance to anoikis, adherence to the pulmonary endothelium, and extravasation into the lung parenchyma (Fig. 6). Our research presented two distinct ways to study OS tumor metastasis within the ACL: (a) an approach of lung-to-lung metastases that required tumor cells to disseminate from left-sided PT and (b) a terminal half of the metastatic process that begins with a direct introduction of cells into the decellularized vascular remnant. Akin to the second method studied in the ACL, the mouse tail vein model provided an additional opportunity to interrogate the latter part of the metastatic cascade. As demonstrated with lung cancer [27], OS PTs within an ACL model are capable of shedding dCTCs from PT, after just 2 days.

Rettew et al. have previously reported that genetic upregulation of EphB2, FGFR2, and RET, which encode RTKs, increases the metastatic potential of tumor cells by enhancing cell proliferation, motility, invasion, and tumorigenicity [31, 32]. Therefore, we expected that the highly metastatic MG63.2 OS cell line would be enriched in those proteins. It was unexpected, however, to discover that dCTCs would coopt many of the same EMT-TFs used by carcinomas. High levels of SNAI2, ZEB1, and TWIST that were also observed (Fig. 4) strongly suggested that OS dCTCs had undergone an EMT-like epigenetic transition.

As stated in the “Introduction,” sarcomas are not epithelial in origin and cannot become more “mesenchymal” [33–35], so we prefer to describe the EMT-like epigenetic effect as dedifferentiation, which describes rejuvenated cell fate as cells shift up the Waddington epigenetic differentiation landscape [64]. Since epithelial and non-epithelial tumors upregulate EMT-TFs as they acquire a less differentiated stem-like state, our team and others suggest a more unifying rubric that emphasizes the EMT-TFs themselves [11]. An added benefit of describing EMT and dedifferentiation by EMT-TFs, rather than by phenotypic markers, is the natural attention to the mechanistic underpinnings of cell fate, whereby EMT-TFs bind gene enhancer and promoter elements to orchestrate epigenetic changes in response to microenvironmental cues.

To further explore the mechanistic relationship between EMT-TFs and stemness, we investigated several proteins of the YAP/TAZ/AXL axis that are widely reported to regulate organ size and mesenchymal cell fate. Their dysregulation has been shown to promote tumor growth and invasion in a wide range of epithelial and non-epithelial malignancies, including carcinomas and sarcoma [39, 40]. The YAP protein, highly expressed in human OS tissues, is more commonly found in advanced clinical stage [65] and may serve as a prognostic indicator [66].

The mechanism of YAP upregulation in OS is complex but appears to interface with or phenocopy the stem cell TF SOX2 through canonical Hippo signaling in response to mechanotransduction [67]. Notably, recent work by Lehmann et al. has shown that ZEB1 and YAP simultaneously bind the same TEAD promoter binding sites of CTGF, CYR61, and AXL [68]. This surprising intersection of the ZEB1-related EMT and YAP-mediated control of mesenchymal cell fate provides a fertile area for further exploration.

Drugs targeting AXL, a downstream effector of YAP/TAZ found in numerous cancer types, are being explored in the preclinical setting [69, 70]. Further, via the ABL2 protein intermediate, AXL can also act upstream of TAZ in some cancer types to promote a feed-forward activation loop amenable to therapeutic intervention [71]. Collectively, our data suggest that the YAP/TAZ/AXL axis contributed to the aggressive behavior of the OS dCTCs by promoting cell stemness, demonstrated by their high expression on ZEB1-, TWIST-, or AXL-related genes. In turn, changes in cell fate led to dCTC dissemination, doxorubicin resistance, and enhanced metastatic potential. Although those effects are well documented in carcinomas [72, 73], our research highlights their contribution to sarcoma dedifferentiation and substantiates a prior finding that TWIST upregulation invokes a dedifferentiated osteoprogenitor-like state [74].

Our OS ACL model provided a unique opportunity to rapidly profile dCTCs and shed new light on molecular drivers of cell differentiation/dedifferentiation and metastasis. For example, AXL blockade was shown to attenuate the proliferation, migration, and metastasis of OS cells. We note that the ACL model itself does not, nor was it intended to, perfectly replicate all aspects of the human lung microenvironment. The residual collagenous capillary network that persists following the detergent-based decellularization lacks endothelial cells.

Despite the advantages of the ACL and rat tail-vein models, additional research is required to determine how well they replicate bone-to-lung hematogenous spread of OS tumor cells. The OS-D/MG63.2 cell lines had been maintained in a non-physiological monolayer environment for years, which could have impacted their plasticity. Though our models lack immune cells and other cellular constituents, they nevertheless supported the development of macroscopic tumors.

In conclusion, our OS ex vivo ACL and in vivo experimental metastasis models mimicked several vital steps required for tumor metastases. Importantly, the ACL model elicited abundant dCTCs that highly expressed EMT-TFs classically associated with EMT or dedifferentiation (Fig. 6). Given the unparalleled ability to experimentally regulate the ACL TME and partially replicate the metastatic cascade, these systems offer a unique opportunity to investigate how microenvironmental cues, growth factors, and other EMT drivers work in concert to regulate tumor cell fate. The strong presence of EMT-related TFs in OS dCTCs and a close connection to the YAP/TAZ mechano-sensing pathway are remarkable findings that may ultimately pave the way for future mechanistic studies that determine why high-grade OS cells are stem-like in nature [35].

## Supplementary information

Supplementary Materials, Methods, Figure Legends X Figures
