# Supplementary material for: Transcriptional activators YAP/TAZ and AXL orchestrate dedifferentiation, cell fate, and metastasis in human osteosarcoma
Source: Cancer Gene Ther. 2021 Jan 6;28(12):1325–38. doi: 10.1038/s41417-020-00281-6 (PMC8636268; doi:10.1038/s41417-020-00281-6)

Supplementary Materials, Methods and Figure Legends

Supplementary Materials and Methods

Cell Culture and Lung harvest

The lung was decellularized by perfusing with heparinized PBS for 15 minutes, 0.1% SDS for 2 hours, water for 15 minutes, and finally with 1% Triton-X-100 for 15 minutes. The acellular lung was washed with 1X antibiotics (Penicillin-Streptomycin-Amphotericin) in PBS for 5 days and stored at -80°C until the next use.

OS cell culture in the *ex vivo* lung model

For studies involving primary tumor growth and treatment, 2.5×10^7 OS-D cells were seeded through the trachea. For studies involving metastasis of OS cells into the lung to form metastatic lesions, 1.0×10^7 cells were seeded in the pulmonary artery. For the studies evaluating the formation of a primary tumor, dCTC and metastatic lesions, we tied off at the right bronchus using silk thread before seeding the tumor cells through the trachea, as described previously. Derived CTCs were isolated from circulating media by centrifugation at 500g/5min at room temperature during media change and enumerated using an automated cell counter (TC20 cell counter, Bio-Rad).

Treatment of OS-D cells on ACL model with doxorubicin

To compare the effect of doxorubicin on OS-D cells grown as monolayer-2D and on dCTC from the 4D lung model, we seeded 25,000 cells/well in triplicate in 96-well plates with 200 μl cultured media and treated it with 3 μM doxorubicin for 48 hours. The control wells with cells received a similar volume of ethanol. After 48 hours, the total live cells were counted using the trypan blue exclusion method.

Histology and Immunohistochemistry analyses

Lung tissues from the MG63.2 xenograft group of mice treated with AXL inhibitor or placebo were fixed in 10% formalin, embedded in paraffin, and then sliced in 5µm sections. The EZ-retriever microwave-based pretreatment and antigen retrieval system (Biogenex, CA) used for dewaxing, rehydration, and antigen retrieval of these FFPE lung tissue sections. AXL, YAP, TAZ, and vimentin protein expression by IHC were evaluated on Leica Bond MAX Auto-stainer by using primary antibodies, rabbit polyclonal antibody anti-human AXL (Cell Signaling Technology, #8661), rabbit polyclonal antibody anti-human YAP (Cell Signaling Technology, #14074), mouse monoclonal antibody anti-human TAZ (Abcam, #ab242313), and mouse monoclonal antibody anti-human vimentin (Leica, #PA0640); respectively. These primary antibodies were detected using Bond polymer refine detection kit according to the manufacturer protocol (Leica, #DS9800). Then, the slides were dehydrated in grade alcohols, cleared in xylene, cover-slipped, and imaged with Keyence Microscope (Keyence, Tokyo, Japan) at 20X resolution. The digital images were processed and quantified using the Visiopharm software version (2020.04) (Hoersholm, Denmark). An APP (Analysis Protocol Package) was designed to quantify cell-based DAB staining in MG63.2 preclinical samples using traditional thresholding methods. The algorithm was built around a three-step approach 1) Pre-Processing: HDAB-DAB and HDAB-Hematoxylin features were used to detect AR Positive cells (Green masking) and Negative cells (Blue masking) respectively by setting pixel values. A median filter of size 5X5 was used for the proper segmentation of cells. 2) Post Processing: Additional steps were designed to enhance the performance of the APP. Change by shape excludes the artifacts; merged cells were separated by separate labels step, and certain clear areas in the cells were filled with fill holes step to fully mask the cells. 3) Output Variable: Mean intensity of each cell and from each sample was extracted and exported into a spreadsheet. Finally, the data were plotted on a Scatter Plot using GraphPad Prism software, version 8 showing the AR Mean Intensity of each cell from all the analyzed samples, and the AR Average mean intensity from each preclinical group of treated mice.

Cell migration (wound healing) assay

MG63 and MG63.2 cells were pre-seeded in 6-well plates at the density of 300×10^3/well. 24 h later, a new 200-μl pipette-tip was used to gently and slowly scratch the cell monolayer across the center of the

wells. The detached cells were gently washed away with culture medium. After replenishing the wells with fresh medium, the gap in the monolayer was captured on Keyence microscope (Keyence, Tokyo, Japan). The scratched cell monolayer was then cultured for another 24 h in the presence of 100nM or 1μM of AXL inhibitor (SGI-7079). The gap in the monolayer was captured again by the same microscope at 9, 15, and 24 h, and the images were analyzed by Image J software (NIH, Bethesda, MD, USA).

RNA Extraction and Quantitative Gene Expression

The total RNA was extracted from monolayer cell cultures, recellularized lung matrices, and collected dCTCs using Isol-RNA lysis reagent (5 PRIME, Gaithersburg, MD, USA), followed by a DirectZol RNA miniprep (Zymo Research, USA) per the manufacturer's instructions. The RNA quality and quantity were determined using Nanodrop 1000 spectrophotometer (Thermo Scientific, Waltham, MA, USA). The cDNA was prepared using a high-capacity cDNA Reverse Transcription kit (Applied Biosystems, NY, USA) with 100–500 ng of total RNA and a real-time PCR assay was performed with sensiFAST SyBR No-ROXreagent (Bioline USA, Taunton, MA). The primers were designed using Primer3Plus online too[1]. Equal amounts of each RNA sample were used as PCR templates in reactions to obtain the threshold cycle (C_t). The C_t was normalized using the known C_t from the housekeeping gene (beta microglobulin) RNAs to obtain ΔC_t. To compare the relative levels of gene expression in different samples (2D, 4D, and CTC), ΔC_t values were calculated by using the ΔC_t values associated with the expression levels in 2D as the basis. ΔC_t values were then transformed to the real fold increase in expression by $2^{\Delta Ct}$. A real fold change was calculated using the $2^{(\Delta\Delta Ct)}$ formula.

Western blot profiling

The protein AXL, pAXL, and GAPDH expression were evaluated using the following antibodies: anti-AXL (#8661, Cell Signaling Technology), anti-pAXL-Y779 (#AF2228, R&D Systems), and anti-GAPDH (#2118, Cell Signaling); respectively. The immune-reactive proteins were captured using horseradish peroxidase-conjugated secondary anti-rabbit IgG or anti-mouse IgG antibodies (Cell Signaling Technology), amplified using the SuperSignal West Dura chemiluminescent substrate (Thermo Fisher Scientific), detected using the Chemi-Doc system (Bio-Rad), and quantified for their densitometry using the ImageJ Gel Analysis tool (NIH, Bethesda, MD).

Immunostaining of OS cells, dCTCs and primary tumors

Monolayer OS-D cell culture in 8 chamber slide or collected dCTCs were fixed for 10 min at room temperature with 4% paraformaldehyde in phosphate-buffered saline (PBS). The primary tumors were harvested, fixed in 10% formalin, embedded in paraffin (formalin-fixed, paraffin-embedded: FFPE), and then sliced in 5 µm sections before processing them for antigen retrieval using 0.1M citrate buffer for 20 minutes and in a vegetable steamer. Fixed dCTCs were smeared across a gelatin-coated slide forming a monolayer of cells. Altogether, monolayer 2D, dCTC, and primary tumor slides were permeabilized and blocked with superblock buffer (Thermo Fisher Scientific, #37535) for 1 hour at room temperature. Slides were then incubated consecutively with primary antibodies to YAP-1 (Santa Cruz, #sc-271134), TAZ (Abcam, #ab84927), AXL (Cell Signaling Technology (CST), #8661), Ret (CST, #14556), Sox9 (CST, #82630), FGFR2 (CST, #23328), N-Cadherin (Biolegend, #350802), TWIST1 (Antibodies-online, #ABIN1724845), ZEB1 (Antibodies-online, #ABIN1724885), EphB2 (CST, #83029) (overnight at 4°C) and Alexa Fluor 488-labeled Goat-anti Rabbit (Thermo Fisher Scientific, #A11037) or Alexa Fluor 568-labeled Goat-anti Mouse (Thermo Fisher Scientific, #A11019) antibodies (for 1hr at room temperature). The nuclei were visualized using Hoechst (Thermo Fisher Scientific, #H357) and the immunofluorescence was acquired after subtracting the background intensities using the Nikon A1-Rsi confocal microscope (Nikon). The fluorescence-labeled for all proteins in both nuclei and cytosol regions was quantified using the Imaris software (Bitplane) and its Cell module that use validated algorithms to define the segmentation by permitting the recognition of selected protein fluorescence in both nuclear and cytosolic regions.

MG63 and MG63.2 OS *in vivo* models and pulmonary metastasis assessment

For experimental lung metastasis experiments, male non-obese diabetic (NOD)-SCID-IL-2Rg^{null}mice (NSG mice; The Jackson Laboratory; Bar Harbor, ME) were used to generate tail vein ($5X10^5$ cells injected/animal) MG63 or MG63.2 xenografts with parental luciferase-expressing MG63 or metastatic MG63.2 cell lines. One day before the IV injection of OS cell lines, the mice were randomized into treatment and placebo groups (3 to 4 mice per group), received a daily single oral gavage dose of SGI-7079 (AXL inhibitor; Selleck) at 100mg/kg or placebo for up to 25 days through a blinding procedure.

Tumor formation was followed by bioluminescence imaging on an IVIS spectrum instrument (Caliper Life Science) after Isoflurane-anesthetization of the animals, IP injection of d-luciferin (PerkinElmer), and quantified with Living Image software (PerkinElmer).
The lungs of NSG mice injected with luciferase-expressing cells were also checked for metastases by counting the number of tumor nodules after Bouin's staining of lungs.

Supplemental Figure Legends

Supplemental Fig. 1. Tumor growth of Osteosarcoma cells on the ex vivo ACL model. A-D: Tumor nodules can be visualized on left lobes (blue arrows) in the ex vivo 4D metastasis model upon injection of 2D cells through the trachea and after 15 days of tumor growth **(A)**. H&E staining of the primary tumor (left lobes) shows a robust growth of tumor cells in the alveolar region **(B)**, while vasculatures (red arrows) were intact without tumor growth inside **(C)**. Right lobes (Metastatic side) of the same lung shows metastatic lesion formation (pink arrows) **(D)**. **E-H**: OS- cells colonize (Metastasize) within three days upon injection of 2D cells through the pulmonary artery **(F and G;** red arrows**)**; however, no visible tumor nodules appeared **(E)**. Massive OS-cell colonization after 15 days of cells injection **(H)**. The number of fields or events analyzed in this figure S1 is 5. Scale bar, 100μm.

Supplemental Fig. 2. The ACL OS model exhibits *in vivo* like sensitivity to doxorubicin. A-D: Visible tumor nodules disappeared after seven days upon 3μM doxorubicin treatment **(A)**. H&E of the primary tumor shows apoptotic and ghost cells (red arrows, **B&C**). No cells were found on the metastatic site, i.e., right lobes **(D)**. Treatment of OS with doxorubicin on the ex vivo 4D model causes tumor regression. Untreated control (**E**) showed higher ki67 positive cells as compared to doxorubicin treatment (**F**) (**E** vs. **F**, p=0.057, **I**), while the apoptotic index (CASP3 IHC stain) was significantly elevated in doxorubicin-treated OS model (**H**) as compared to untreated control (**G**) (**G vs. H**, p<0.0001, J). The number of cells in the high-power field (HPF) in the primary tumor (left lobe) and metastatic tumors (right lobe) were compared between doxorubicin treated vs. untreated control, and it was significantly low upon treatment (**K & L**). CTCs obtained from the model showed a mild effect of doxorubicin in 96-well plate set up for 48 hrs (p=0.075, **N**). In contrast, 2D cells showed significantly less live cells after 48 hrs of doxorubicin treatment in 96 well set up (p<0.0001, **M**). The number of fields or events analyzed in this figure S2 is 5. Scale bar, 100μm.

Supplemental Fig. 3. Derived CTCs (dCTC) have downregulated non-active cytoplasmic form of the principal effectors of the Hippo pathway, YAP/TAZ, and AXL. A) Representative YAP-1, TAZ, and AXL immunofluorescence confocal microscopy staining quantifications within the cytoplasmic single cells derived from OS-D-2D, PT, and dCTC samples. Scatter plots represent the mean value of 3 experiments for OS-D 2D-monolayer cultures and four experiments for the PT and dCTC. Bars represent standard deviations. PT= primary tumor; dCTC=derived circulating tumor cell. **B)** CTCs derived from the *ex vivo* 4D ACL model have significantly higher AXL gene expression, as compared by qRT-PCR analyses to respective primary tumor nodules formed on this model and OS-D cells grown as 2D-monolayer. Columns represent the mean values of n=3 experiments, and bars represent standard deviations.

Supplemental Fig. 4. Derived CTCs (dCTCs) have an enhanced stemness phenotype. A) CTCs derived from the *ex vivo* 4D ACL model have significantly higher Snail2 gene expression, as compared to respective primary tumor nodules formed on this model and OS-D cells grown as 2D-monolayer. No difference between PT and dCTC in N-cadherin gene expression. Columns represent the mean values of n=3 experiments, and bars represent standard deviations. **B)** Representative N-Cadherin immunofluorescence confocal microscopy staining and quantification**, C)** within the single cell or**, D)** the averaged OS-D-2D, PT, and dCTC samples. 20μm bares are shown. Scatter plots represent the mean value of 3 experiments for OS-D 2D-monolayer cultures and four experiments for the PT and dCTC. Bars represent standard deviations. Scale bar, 20μm. PT= primary tumor; dCTC=derived circulating tumor cell.

Supplemental Fig. 5. AXL inhibition attenuates the proliferation of OS cell lines. A) Profiling of MG63 and MG63.2 OS cells for AXL protein expression by immunofluorescence analysis, and quantitative representation of the AXL mean nuclear as well as the mean cytoplasmic intensities. Bars show mean ± SD. **B)** MG63.2 cells are more sensitive to SGI-7079 than MG63 cells, as shown by the in vitro WST1-Proliferation cell-based assay. **C)** SGI-7079 inhibits Gas6-induced AXL phosphorylation, as demonstrated by western blot analysis in MG63 and MG63.2 2D-monlayer cell

cultures. **D)** Normalized AXL and pAXL expression relative to GAPDH within the same samples presented in the Figure 5C.

Supplemental Fig. 6. AXL inhibition attenuates the migration of OS cell lines. Wound healing assay using MG63 and MG63.2 cells: 9, 15, and 24 h post-injury wound healing was evaluated in untreated cells and in cells treated with 100 nM AXLi, SGI-7079, as indicated.

Supplemental Fig. 7. Immunohistochemical evaluation of vimentin, AXL, YAP, and TAZ expression in OS MG63.2 pulmonary metastases after placebo and AXL blockade treatments. A) IHC stains for AXL, YAP, TAZ and vimentin in lung tissues of all MG63.2 xenograft mice after placebo or AXLi treatments. 100 μm scale bars are shown. **B)** Representative IHC vimentin, AXL, YAP, and TAZ mean intensity quantification of the preclinical OS MG63.2 pulmonary metastases after placebo and AXL blockade treatments, within the single cell or, **C)** the averaged tumor cell samples (placebo and AXLi). Bars represent standard deviations.

Reference

1. Baba AI, Catoi C. Comparative Oncology. Bucharest (RO)2007.

Supplemental Figure 1

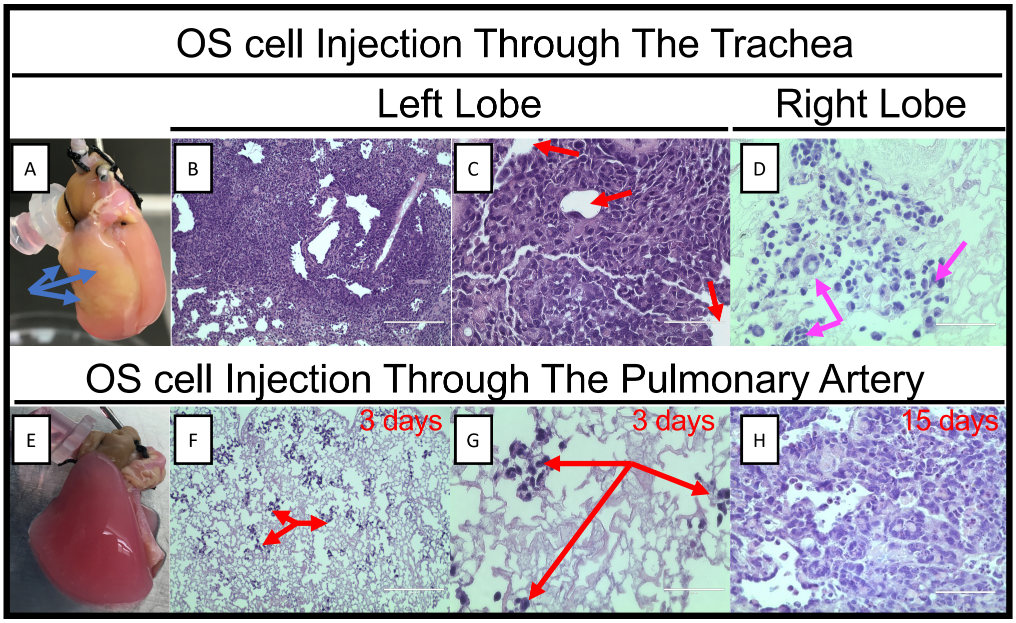

Supplemental Figure 2

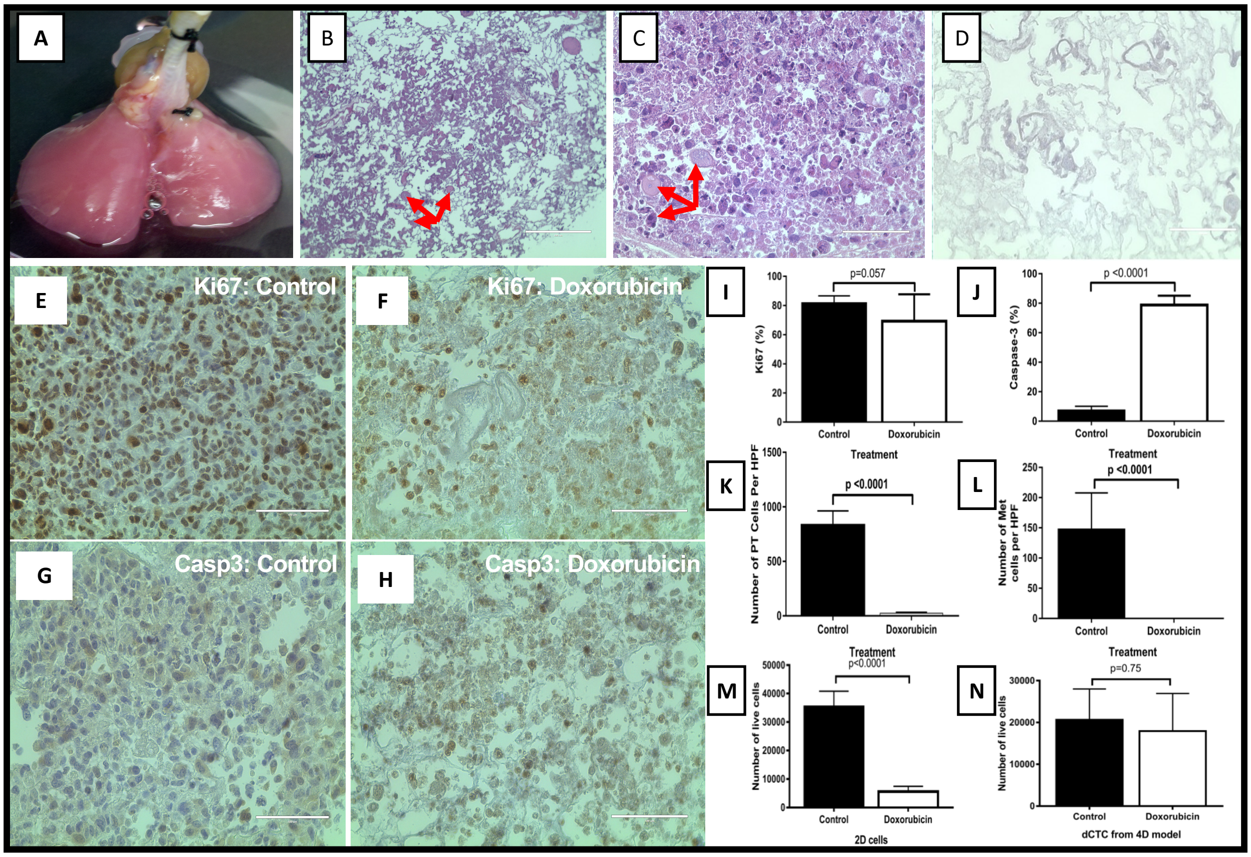

Supplemental Figure 3

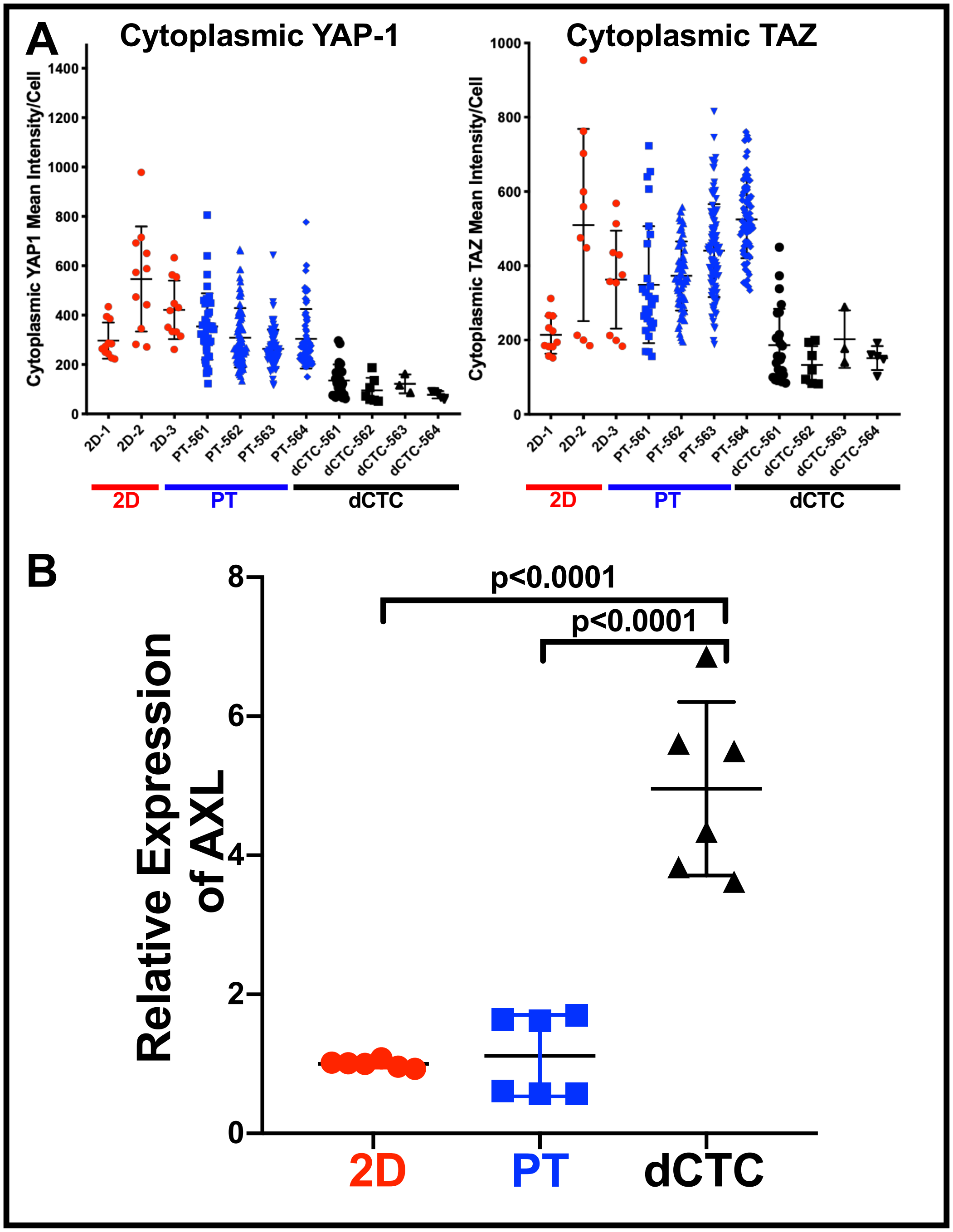

Supplemental Figure 4

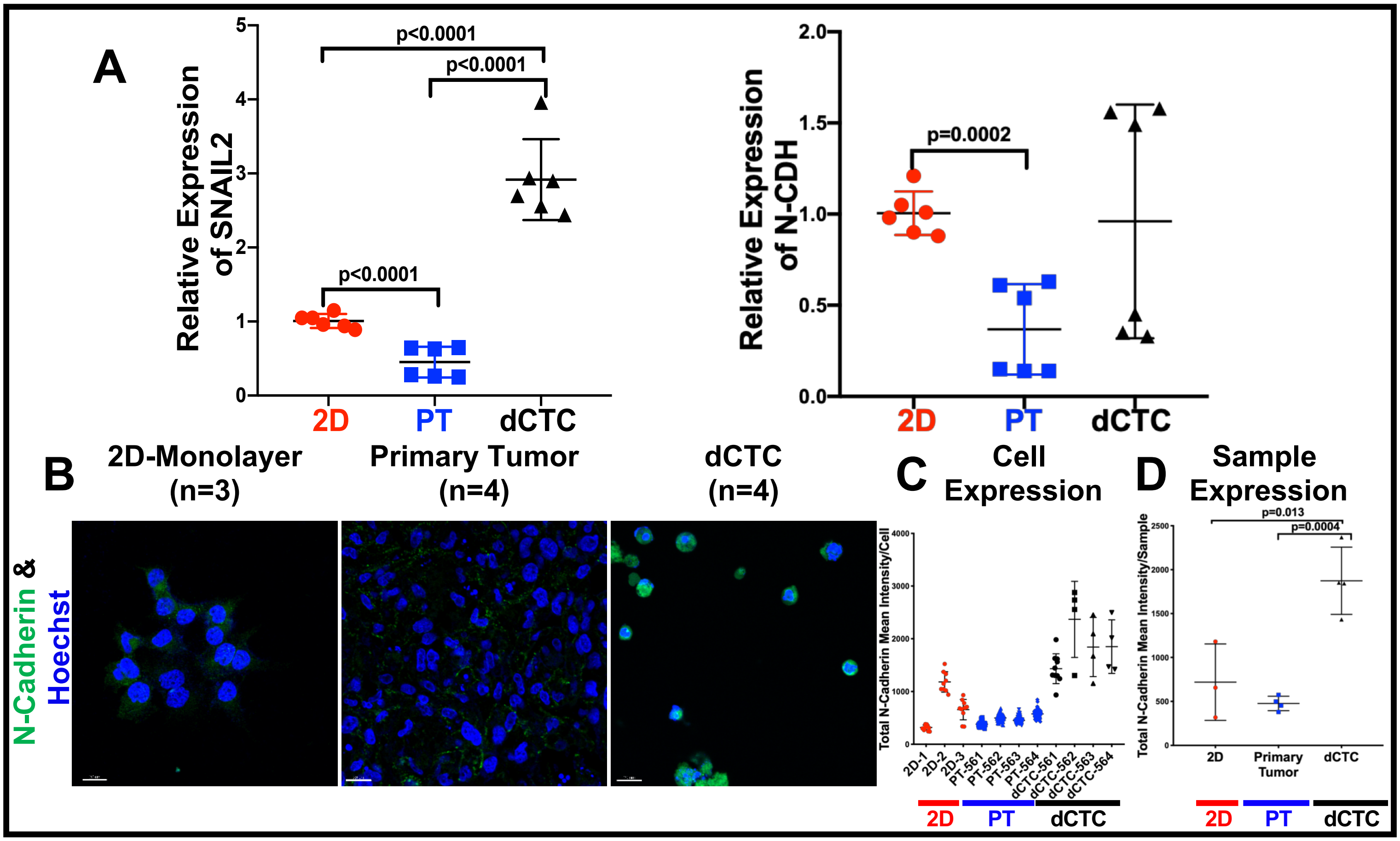

Supplemental Figure 5

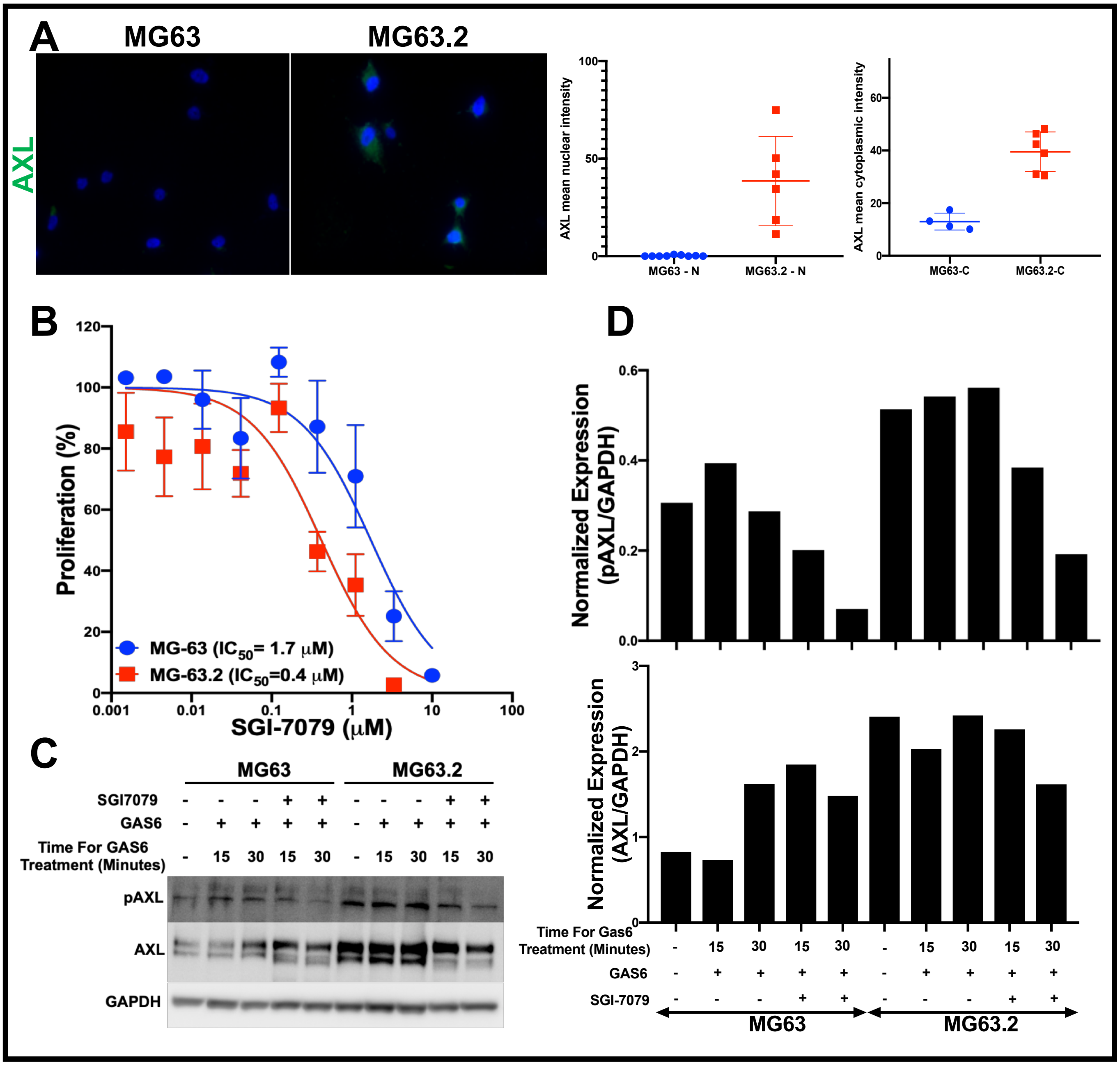

Supplemental Figure 6

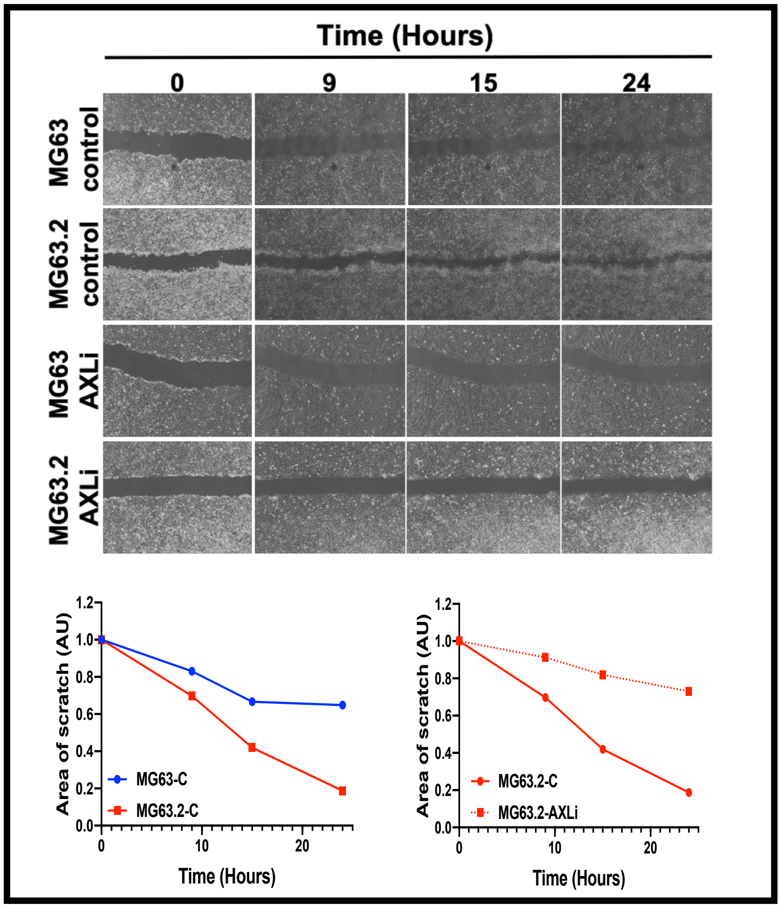

Supplemental Figure 7

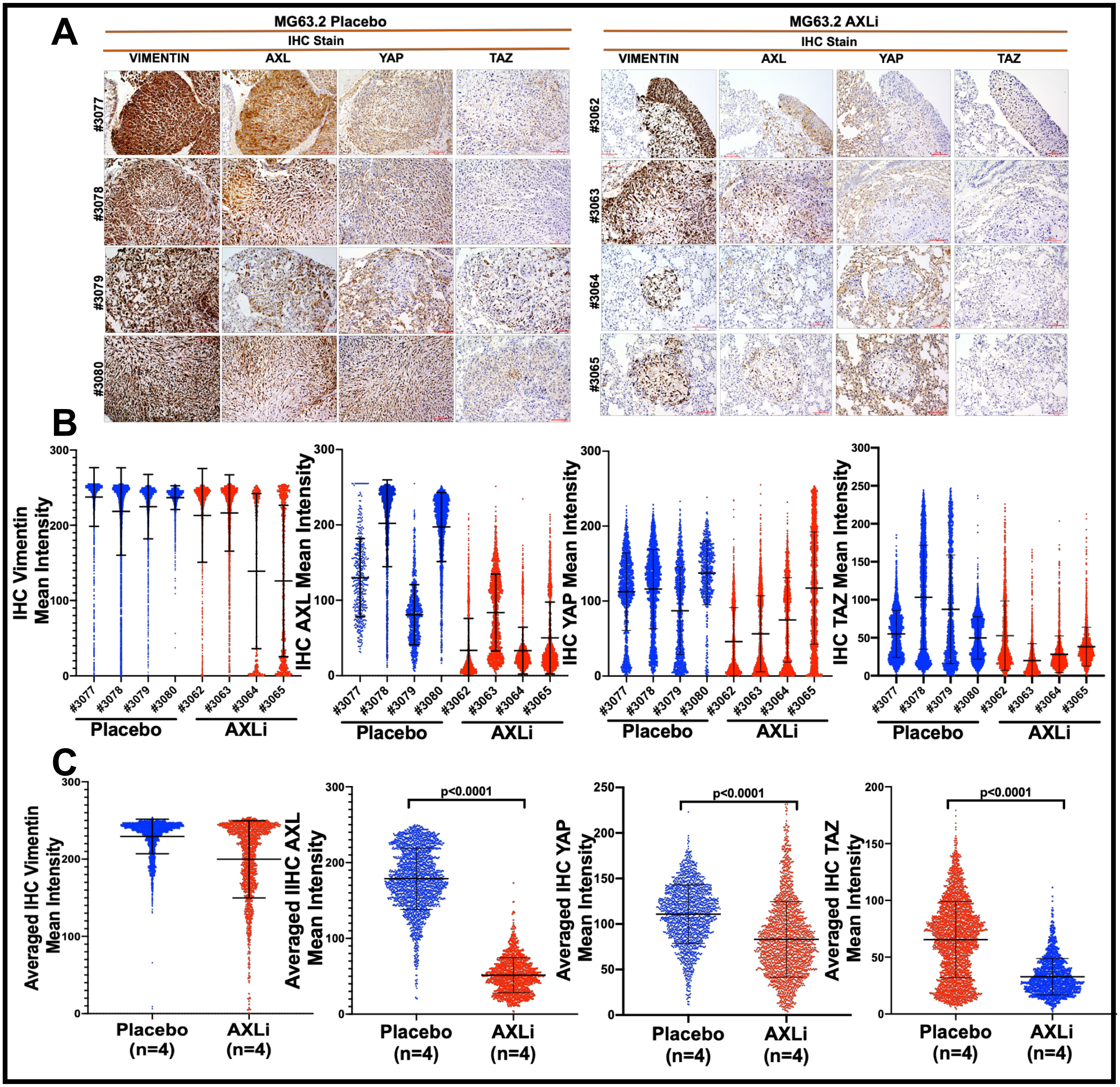

Supplement: Supplementary file 1 — Supplementary Materials, Methods, Figure Legends X Figures [file 41417_2020_281_MOESM1_ESM.pdf]
